# Oxidation-Shielded *P*(*St-MMA*)*@Fe*_3_*O*_4_*@P*(*St-MMA*) Mesoporous Magnetic Microspheres: A Robust Solid-Phase Carrier for Ultrasensitive CEA Chemiluminescence Immunoassay

**DOI:** 10.3390/bios16060303

**Published:** 2026-05-22

**Authors:** Yu Chen, Lina Dong, Hengyan Tian, Fei Yang, Dengbang Jiang, Minglong Yuan

**Affiliations:** National and Local Joint Engineering Research Center for Green Preparation Technology of Biobased Materials, Yunnan Minzu University, Kunming 650500, China

**Keywords:** magnetic microspheres, chemiluminescence enzyme immunoassay (CLEIA), carcinoembryonic antigen (CEA), sandwich structure, oxidation resistance, mesoporous architecture

## Abstract

Magnetic polymeric microspheres are pivotal solid-phase carriers in chemiluminescence enzyme immunoassays (CLEIA). However, their practical clinical application is frequently hindered by non-specific adsorption, irreversible aggregation, and the intrinsic susceptibility of exposed outermost Fe_3_O_4_ nanoparticles to oxidation. To overcome these critical bottlenecks, we rationally engineered highly original monodisperse *P*(*St-MMA*)*@Fe*_3_*O*_4_*@P*(*St-MMA*) sandwich-structured microspheres. The bespoke amphiphilic outer shell acts as an impenetrable shield against hydration and oxidation, while maintaining a topologically size-matched mesoporous network (average pore size of 13.11 nm) for optimal antibody anchoring. Strikingly, this architecture ensures exceptional long-term colloidal stability, completely preventing macroscopic agglomeration for over six months in buffer solutions. When evaluated in a carcinoembryonic antigen (CEA), CLEIA, our microspheres achieved an ultra-low limit of detection (LOD) of 0.055 ng·mL^−1^ and high analytical recovery (93.37–108.25%). In a head-to-head comparison with industry-standard commercial magnetic beads, the engineered microspheres delivered stronger chemiluminescent signals and lower background noise, demonstrating excellent intra-assay (CV < 4.37%) and inter-assay (CV < 10%) precision. This work establishes a scalable, highly stable materials platform that effectively resolves the persistent oxidation limitations, holding immense practical importance for next-generation ultrasensitive clinical in vitro diagnostics.

## 1. Introduction

Chemiluminescence enzyme immunoassay (CLEIA) has emerged as a mainstream technique in the field of in vitro diagnostics (IVD) owing to its exceptional sensitivity, high stability, broad dynamic range, and amenability to full automation [1,2,3,4]. As the core solid-phase carrier in CLEIA systems, magnetic microspheres are responsible for capturing antigen-enzyme-antibody immune complexes and enabling rapid magnetic separation from free interferents [5,6,7,8]. Consequently, the physicochemical properties of these microspheres—such as their sedimentation rate, size distribution, specific surface area, and magnetic responsiveness—directly dictate the ultimate detection efficiency and assay reliability. Among various candidates, magnetic polymeric microspheres, typically composed of *Fe*_3_*O*_4_ nanoparticles integrated with a polymer matrix, have attracted immense attention. They uniquely combine the robust magnetic separation capability of *Fe*_3_*O*_4_ with the excellent biocompatibility, structural stability, and versatile surface functionalization of polymers [9,10,11,12].

To date, diverse strategies have been explored to fabricate functional magnetic polymeric microspheres. Polystyrene (PS) and its derivatives are frequently employed as the polymer matrix due to their outstanding monodispersity and highly controllable particle size [13,14,15]. For instance, Xin et al. prepared $Fe_{3}O_{4}@PS$ core-shell microspheres via micro-suspension emulsion polymerization, achieving surface functionalization through post-sulfonation [14]. Alternative complex architectures, such as phase-inversion composite microspheres with embedded $Fe_{3}O_{4}$ [16] and bowl-shaped PS microspheres with in situ coated $Fe_{3}O_{4}$ nanoparticles [17], have also been developed. In the specific context of CLEIA, sandwich-type magnetic microspheres [18] and well-defined poly(glycidyl methacrylate) (PGMA) microspheres [19,20] have been deliberately synthesized to immobilize proteins and subsequently lower background signals. Superparamagnetic poly(styrene-divinylbenzene-acrylamide) microspheres have also been utilized for multiplexed fluoroimmunoassays [21].

Despite these notable advances, the practical application of existing magnetic polymeric microspheres in CLEIA is frequently hindered by several critical bottlenecks. Conventional microspheres still suffer from severe non-specific adsorption, low specific protein-binding capacity, and broad size distribution. More importantly, conventional fabrication techniques often leave $Fe_{3}O_{4}$ nanoparticles exposed on the outermost layer, making them highly susceptible to irreversible aggregation, oxidation, or detachment in aqueous buffer systems, which severely compromises long-term colloidal stability.

To provide a clearer perspective on the current technological landscape, Table 1 summarizes the key characteristics of recently reported magnetic polymeric microspheres utilized in immunoassay applications. As indicated, while conventional architectures such as $Fe_{3}O_{4}@PS$ core-shell or standard embedded structures possess reliable initial magnetic responsiveness, they frequently lack a defined mesoporous architecture (which is crucial for high-capacity antibody loading) and exhibit limited long-term stability due to poor oxidation resistance. In contrast, developing a solid-phase carrier that simultaneously achieves a large accessible pore size, absolute shielding against aqueous oxidation, and exceptional long-term colloidal stability remains a significant challenge.

To address these formidable challenges, we herein propose a facile yet highly tunable strategy to fabricate monodisperse magnetic polymeric microspheres with a well-defined $P(\mathrm{St}\text{-}\mathrm{MMA})@Fe_{3}O_{4}@P(\mathrm{St}\text{-}\mathrm{MMA})$ three-layer sandwich structure. We systematically investigate the fundamental effects of alkaline hydrolysis degree, buffer pH, and dispersant variations on the $Fe_{3}O_{4}$ coating efficiency over the P(St-MMA) cores. To rigorously validate their practical utility, the optimized sandwich-structured microspheres were directly substituted for the commercial magnetic bead component in a carcinoembryonic antigen (CEA) CLEIA diagnostic kit, allowing for a comprehensive evaluation of their bioconjugation performance and colloidal stability in realistic clinical assay environments.

## 2. Experimental Section

### 2.1. Materials

Styrene (St, 99%), methyl methacrylate (MMA, 99%), methacrylic acid (MAA, 99%), acrylic acid (AA, 99%), and iron(II) chloride tetrahydrate (FeCl_2_·4H_2_O, 98%) were purchased from Shanghai Titan Scientific Co., Ltd., Shanghai, China. Azobisisobutyronitrile (AIBN, 99%, recrystallized) was obtained from Macklin, Shanghai, China). Ethanol, polyvinylpyrrolidone (PVP, MW 45,000–58,000, K30), 2-morpholinoethanesulphonic acid (MES, 98%, Leyan, Nanjing, China), N-(3-dimethylaminopropyl)-N′-ethylcarbodiimide hydrochloride (EDC·HCl, 99%, Yuanye, Shanghai, China), NaOH (98%), NaCl (99%, Sinopharm, Beijing, China), iron(III) chloride hexahydrate (FeCl_3_·6H_2_O, 99%, Aladdin, Shanghai, China), HCl (36–38%, Chuandong Chemical, Chongqing, China), ammonia solution (25%, Zhiyuan Chemical, Henan, China), sodium tripolyphosphate (Na_5_P_3_O_10_, 98%), and ammonium carbonate ((NH_4_)_2_CO_3_, 40%) were all used as received without further purification. The CEA CLEIA kit was provided by Kunming Sun Biomedical Technology Co., Ltd., Kunming, China.

### 2.2. Synthesis of Carboxyl Poly(St-co-MMA) Microspheres

**Figure 1:**
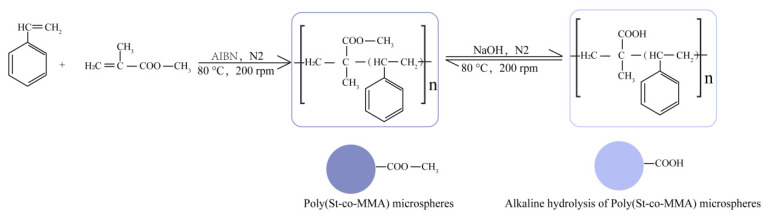
Schematic illustration of the preparation of carboxyl-functionalized poly(St-co-MMA) microspheres.

The monodisperse P(St-MMA) microspheres were synthesized via a dispersion polymerization method optimized in our previous study (Figure 1). Briefly, 12 g of PVP-K30 and 1.5 g of AIBN were completely dissolved in 640 g of 75% ethanol. Subsequently, 30 g of St (0.288 mol) and 28.84 g of MMA (0.288 mol) were introduced into the mixture. Under a continuous nitrogen atmosphere, the reaction was mechanically stirred at 200 rpm, pre-polymerized at 50 °C for 30 min, and then refluxed at 80 °C for 30 h. After polymerization, the resulting microspheres were collected by centrifugation (8000 rpm for 2 min) and washed three times with deionized (DI) water. To introduce carboxyl functional groups, the harvested P(St-MMA) microspheres were subjected to alkaline hydrolysis. Various concentrations of NaOH solutions were added to the microspheres to conduct a hydrothermal reaction at 80 °C for 30 h. The alkali-treated microspheres were washed with DI water until neutral. Afterwards, an equal mass of HCl was added for a subsequent hydrothermal reaction at 80 °C for 30 h. Finally, the hydrolyzed carboxyl P(St-MMA) microspheres were washed to neutrality and dried in a blast oven at 45 °C.

### 2.3. Preparation of Magnetic Polymeric Microspheres

**Figure 2:**
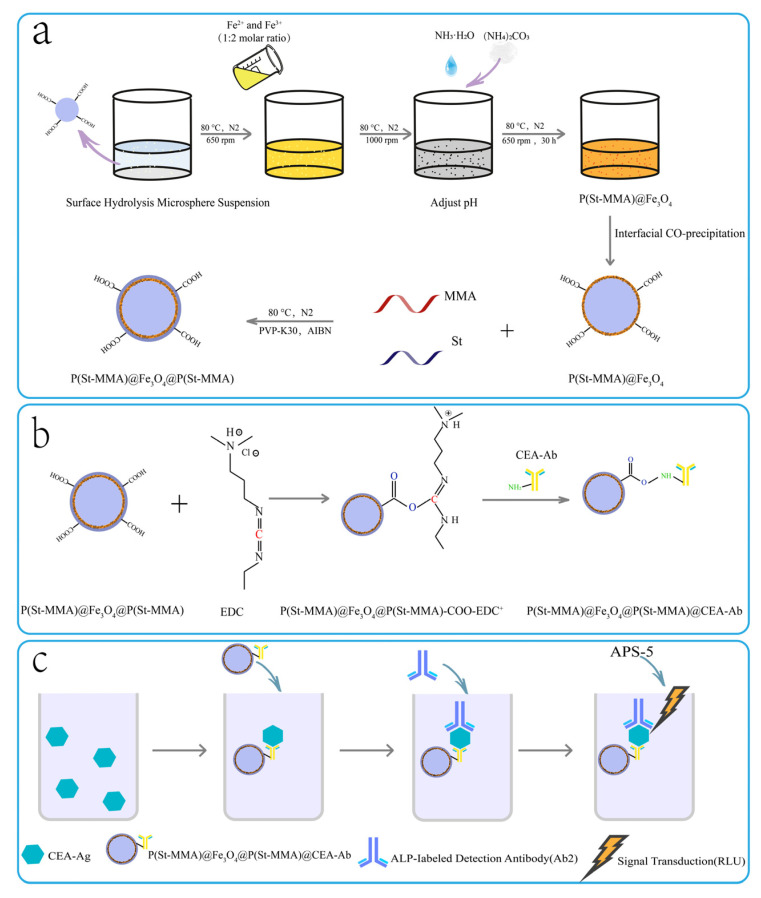
Comprehensive schematic illustration of the materials design and clinical application: (**a**) the complete formation process of the sandwich-structured $P(St\text{-}MMA)@Fe_{3}O_{4}@P(St\text{-}MMA)$ magnetic polymeric microspheres, (**b**) the bioconjugation process of the capture antibody (CEA-Ab) onto the functionalized microsphere surface, and (**c**) the sensing and signal transduction mechanism of the chemiluminescence enzyme immunoassay (CLEIA) for quantitative CEA detection.

The complete formation process of the sandwich-structured magnetic microspheres is schematically depicted in Figure 2a and involves two sequential steps: inner magnetic layer deposition and outer polymeric shell encapsulation. First, to deposit the $Fe_{3}O_{4}$ magnetic layer, 2 g of the alkaline-hydrolyzed P(St-MMA) microspheres were dispersed in 200 g of DI water and ultrasonicated for 30 min before being transferred to a four-neck flask maintained at 80 °C. Mechanistically, the abundant carboxylate (−COO^−^) groups generated on the surface during the prior alkaline hydrolysis serve as dense, homogeneous nucleation sites. These functional sites strongly coordinate with the introduced iron precursor ions ($Fe^{2+}$ and $Fe^{3+}$), strictly guiding the uniform in situ co-precipitation and anchoring of $Fe_{3}O_{4}$ nanoparticles onto the P(St-MMA) core, rather than forming uncontrolled bulk agglomerates in the solution. Concurrently, a 50 mL yellow precursor solution containing $FeCl_{2}\cdot 4H_{2}O$ and $FeCl_{3}\cdot 6H_{2}O$ at a 1:2 molar ratio (designed for a 20–29% magnetic content) was prepared. This precursor was poured into the flask under $N_{2}$ protection and stirred at 650 rpm for 15 min to yield a uniform orange suspension. The stirring speed was then increased to 1000 rpm, and ammonia solution (25%) was added dropwise over 30 min to adjust the pH to 9–14, followed by the addition of the selected dispersant. The blackened mixture was allowed to react at 650 rpm for 30 h at 80 °C, gradually turning reddish-brown. After cooling to room temperature, the obtained $P(St\text{-}MMA)@Fe_{3}O_{4}$ microspheres were magnetically separated, washed repeatedly with MES buffer (pH = 5.5) for neutralization, and stored at 4 °C. In the second step, to form the protective outer layer, 0.5 g of the $P(St\text{-}MMA)@Fe_{3}O_{4}$ microspheres were utilized. Free radical polymerization was initiated on the magnetic surface using AA, St, or MMA monomers to encapsulate the microspheres with a distinct outer layer (PAA, PMMA, or P(St-MMA)), respectively. This process ultimately yielded the target $P(St\text{-}MMA)@Fe_{3}O_{4}@P(St\text{-}MMA)$ three-layer magnetic polymeric microspheres. This outer amphiphilic polymeric shell is strategically designed to act as a robust thermodynamic and physical barrier. Specifically, the hydrophobic styrene (St) segments effectively repel the permeation of surrounding water molecules, while the dense polymer network restricts the diffusion of dissolved oxygen. This hermetic encapsulation physically “locks” the intermediate $Fe_{3}O_{4}$ layer, fundamentally preventing progressive oxidation and ensuring exceptional colloidal stability during long-term storage.

### 2.4. Material Characterizations

The surface morphologies of the microspheres were observed using a scanning electron microscope (SEM, NOVA NANO SEM-450, FEI, Hillsboro, OR, USA) operating at an accelerating voltage of 10 kV with a magnification of 25,000×. Prior to SEM observation, samples dispersed in DI water were cast onto silicon wafers, dried at 45 °C for 5 h, and sputtered with gold. The particle size and polydispersity index (PDI) were determined via wet analysis using a multifunctional particle size and shape analyzer (S3500SI, Microtrac, Lehigh Valley, USA). Chemical functional groups were identified using an infrared (IR) spectrometer (Nicolet Avatar 360, Thermo Nicolet, Madison, WI, USA). The thermal properties and glass transition behavior were evaluated using a differential scanning calorimeter (DSC 214 Polyma, NETZSCH, Selb, Bavaria, Germany) under a nitrogen flow. Briefly, 5–10 mg of the sample was heated from room temperature to 500 °C at a rate of 10 ${{}^{\circ}C\cdot min}^{-1}$, and data were processed using Proteus^®^ 7.0 software. Nitrogen adsorption-desorption isotherms were recorded at 77 K using a specific surface area and pore size analyzer (BELSORP-max, Microtrac MRB, Osaka, Japan) over a relative pressure range of P/P0 = 1 × 10^−9^ to 0.997 to determine the specific surface area, pore volume, and pore size distribution. The colloidal sedimentation performance was assessed by monitoring the absorbance decline at 560 nm (OD560) using a microplate reader (Multiskan GO, Thermo Fisher, Waltham, MA, USA). Microsphere suspensions (0.5 ${mg\cdot mL}^{-1}$, 3 mL) were measured at 0, 5, 10, 15, and 20 min, and the percentage of absorbance decrease was calculated. The surface charge and colloidal stability of the microspheres at different fabrication stages were evaluated by measuring the Zeta potential using a dynamic light scattering (DLS) analyzer (Zetasizer Nano ZS, Malvern Instruments, Malvern Panalytical, Malvern, UK). Prior to the measurements, the microsphere samples were appropriately diluted in deionized water (pH ≈ 7.0) and mildly ultrasonicated for 1 min to ensure uniform dispersion. All measurements were performed in triplicate at 25 °C, and the average values were recorded. The magnetic properties and hysteresis loops of the samples were recorded at room temperature (300 K) using a vibrating sample magnetometer (VSM, 7404, Lake Shore, Westerville, OH, USA) under an applied magnetic field sweeping from −20,000 to +20,000 Oe.

### 2.5. Antibody Coupling and CLEIA Performance Evaluation

The bioconjugation capacity and practical clinical detection performance of the prepared magnetic polymeric microspheres were systematically evaluated using a double-antibody sandwich CLEIA method. The bioconjugation process of the capture antibody onto the functionalized microspheres is explicitly illustrated in Figure 2b. Initially, 10 mg of the microspheres were washed three times with 1 mL of MES buffer (0.1 ${mol\cdot L}^{-1}$, pH = 5.5) under magnetic separation. For carboxyl activation, 1 mL of EDC solution (1 ${mg\cdot mL}^{-1}$ in 0.1 ${mol\cdot L}^{-1}$ MES, pH = 5.5) was added and reacted at room temperature for 0.5 h on a hematology mixer, followed by the magnetic removal of excess EDC. Subsequently, 1 mL of the capture antibody solution (0.16 ${mg\cdot mL}^{-1}$ in 0.1 ${mol\cdot L}^{-1}$ MES, pH = 5.5) was added to the activated microspheres and incubated for 3 h at room temperature. Unreacted antibodies were discarded via magnetic separation. To prevent non-specific binding, the antibody-coated microspheres were blocked with 1 mL of BSA solution (0.01 ${mol\cdot L}^{-1}$ PBS, pH = 7.2, containing 0.5% BSA) at 4 °C for 12 h. After washing three times with TBS-T buffer (pH = 7.2), the bioconjugated microspheres were resuspended in 1 mL of PBS and stored at 4 °C.

For analytical validation, a working solution of the prepared antibody-coated microspheres (0.3 ${mg\cdot mL}^{-1}$) was formulated and used to directly substitute the commercial magnetic bead component in the CEA chemiluminescence kit (Kunming Sun Biomedical Technology Co., Ltd., Kunming, China). The overall sensing and signal transduction mechanism of the double-antibody sandwich CLEIA is systematically illustrated in Figure 2c. The immunoassay was performed using a fully automated CLEIA analyzer (Shenzhen Yingkai Biotechnology Co., Ltd., SHINE i2900, Shenzhen, China). The magnetic microspheres specifically captured the CEA antigen and enzyme-labeled antibody to form an immune complex. Following the magnetic separation of free interferents and the addition of the chemiluminescent substrate, the relative light units (RLU) of the CEA calibrators were quantitatively determined via the analyzer’s photomultiplier tube.

## 3. Results and Discussion

### 3.1. Structural Evolution and Thermal Properties of Hydrolyzed P(St-MMA) Microspheres

**Figure 3:**
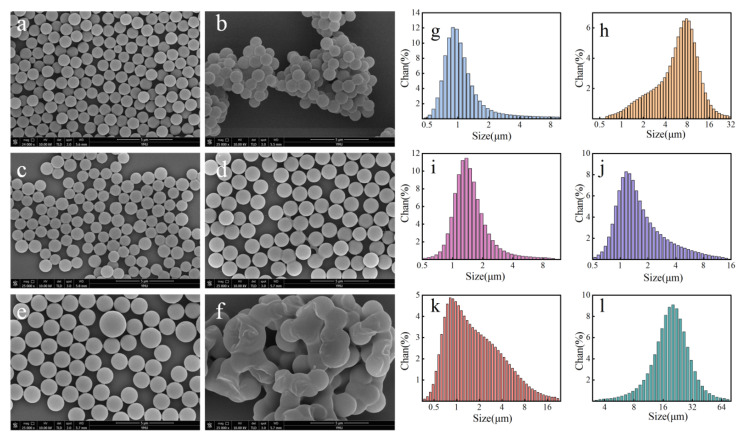
SEM images (**a**–**f**) and corresponding size distributions (**g**–**l**) of pristine P(St-MMA) and hydrolyzed P(St-MMA) microspheres under different hydrolysis conditions: (**a**,**g**) P(St-MMA), (**b**,**h**) P(St-MMA−1), (**c**,**i**) P(St-MMA−2), (**d**,**j**) P(St-MMA−3), (**e**,**k**) P(St-MMA−4), (**f**,**l**) P(St-MMA−4).

The morphological and structural evolution of P(St-MMA) microspheres following alkaline hydrolysis is illustrated by the SEM images and corresponding size distributions in Figure 3 and Table 2.

Pristine P(St-MMA) microspheres exhibited a highly uniform spherical morphology with an average diameter of 0.95 ± 0.001 μm. Upon alkaline treatment, the ester groups on the polymethyl methacrylate (PMMA) side chains underwent partial hydrolysis, converting into poly(methacrylic acid) (PMAA). Consequently, the ionization of these newly generated hydrophilic carboxylate groups induced significant water uptake and subsequent swelling of the microspheres. In this study, the number of hydrolysis cycles was identified as a critical determinant of colloidal stability and surface functionality. A single hydrolysis cycle at 13 ${mol\cdot L}^{-1}$ NaOH (Sample P(St-MMA)−1) resulted in severe microsphere agglomeration and a broadened PDI. This is likely attributed to insufficient surface hydrolysis, which failed to generate a strong enough electrostatic repulsion to counteract the van der Waals forces, leading to inter-particle bridging.

Interestingly, increasing the frequency of alkaline treatment significantly improved the colloidal quality. After three consecutive hydrolysis cycles (Samples P(St-MMA)−2 and P(St-MMA)−3), the microspheres reached an optimal swelled diameter of approximately 1.3 μm while regaining excellent monodispersity (PDI < 0.1). Specifically, P(St-MMA)−3 was selected as the optimal substrate for subsequent magnetic loading due to its superior balance of surface carboxyl density and structural integrity. However, as the treatment proceeded to four cycles (Sample P(St-MMA)−4), the PDI sharply increased to 1.46, marking it as a failed control case where over-hydrolysis induced irreversible colloidal destabilization and massive clustering. Under even more extreme alkaline conditions (33 ${mol\cdot L}^{-1}$, Sample P(St-MMA)−5), the polymer cross-linking network was completely disrupted, triggering total structural collapse and fusion into large aggregates (~18.61 μm).

Fourier transform infrared (FTIR) spectroscopy was performed to track the chemical modifications and identify the functional groups of the microspheres. A detailed assignment of the characteristic FTIR bands is summarized in Table 3. As depicted in Figure S1, all samples exhibited broad O−H stretching vibrations around 3436 $cm^{-1}$ (arising from the carboxyl groups or adsorbed water), distinct aromatic and aliphatic C−H stretching (3023, 2944, 2851 $cm^{-1}$), and a sharp, dominant peak at 1734 $cm^{-1}$ corresponding to the C=O stretching of the ester groups in the MMA segments. Additionally, the characteristic aromatic ring skeleton vibrations of styrene were clearly observed at 1598, 1498, and 1451 $cm^{-1}$. Notably, the characteristic peak positions and relative intensities remained largely analogous before and after alkaline treatment. This phenomenon can be thoroughly explained by the mechanisms of the base-catalyzed hydrolysis and the detection limits of bulk FTIR. During the hydrothermal reaction, the hydroxide ions ($OH^{-}$) execute a nucleophilic attack on the electrophilic carbonyl carbons of the exposed ester groups, cleaving the methoxy groups to generate carboxylate functions, which subsequently convert to poly(methacrylic acid) (PMAA) upon neutralization. However, this hydrolysis process is predominantly a surface-confined reaction restricted to the outermost nanometer layers of the microspheres. Because standard FTIR spectroscopy probes the bulk material with a penetration depth in the micrometer range, the overwhelming majority of the signals still originate from the intact, unhydrolyzed P(St-MMA) bulk cores. Therefore, the massive ester C=O peak at 1734 $cm^{-1}$ heavily overshadows the relatively small population of the newly formed PMAA carboxyl C=O peaks (which typically appear around 1700–1710 $cm^{-1}$ and overlap with the ester peak). Nevertheless, the successful localized formation of the PMAA segments is unambiguously validated by the thermal dynamic alterations observed in the subsequent DSC analysis.

DSC further unraveled the thermal dynamic alterations induced by the alkaline treatment (Figure S2 and Table 4). The pristine P(St-MMA) microspheres displayed a single glass transition temperature ($T_{g}$) at 95.1 °C, aligning with typical PMMA/PS blocks. After controlled hydrolysis (Samples P(St-MMA)−2, −3, and −4), a distinct second $T_{g}$ emerged between 174 °C and 194 °C. Since the theoretical $T_{g}$ values for bulk PMMA and PMAA are approximately 90 °C and 170 °C, respectively, the emergence of this high-temperature transition unambiguously confirms the successful conversion of PMMA side chains into PMAA domains. Notably, under excessive alkaline treatment (Sample P(St-MMA)−5), the dual $T_{g}$ vanished, yielding a single, much sharper peak shifted to 119.3 °C. Mechanistically, while moderate $OH^{-}$ attacks the electrophilic carbonyl carbons to yield PMAA, an excessive presence of $Na^{+}$ readily neutralizes the carboxyl groups to form sodium carboxylate ionomers. These strong ionic interactions act as physical cross-linking points, severely restricting the segmental mobility of the polymer chains and ultimately elevating the required thermal energy for the glass transition.

To gain deeper insights into the macromolecular architecture and phase behavior post-hydrolysis, the thermodynamic transitions observed in the DSC curves (Figure S2 and Table 4) warrant a detailed mechanistic discussion. The pristine P(St-MMA) copolymer exhibits a single, well-defined glass transition temperature ($T_{g}$) at 95.1 °C, indicating a homogeneous amorphous phase. Strikingly, upon alkaline hydrolysis, the copolymer shifts toward a distinct dual-$T_{g}$ behavior (for samples P(St-MMA)−2, −3, and −4).

This thermal transition duplication is fundamentally governed by the base-catalyzed conversion of ester groups into hydrophilic MAA or sodium methacrylate ions, which drives localized microphase separation due to thermodynamic incompatibility between the blocks. The lower transition temperature ($T_{g1}$, ~105 °C) is closely associated with the cooperative segment relaxation within the unhydrolyzed hydrophobic P(St-MMA) domains. Conversely, the newly emerged, exceptionally high transition temperature ($T_{g2}$, ranging from 174.3 °C to 194.17 °C) provides indisputable evidence for the formation of an ionomeric matrix layer. According to the classic Eisenberg-Hird-Moore (EHM) ionomer model [22], the highly polar carboxyl groups and carboxylate ions (−COO^−^Na^+^) spontaneously aggregate into localized, nanometer-scale multiplets or ionic clusters. These clusters act as robust physical crosslinking networks that severely restrict the rotational freedom and local mobility of the surrounding polymer backbones. Consequently, a much higher thermal energy input is required to trigger the glass transition relaxation within these structurally locked ionic domains.

Furthermore, as the degree of hydrolysis intensifies from P(St-MMA)−2 to P(St-MMA)−4, the density of these ionic aggregates escalates, leading to a predictable upward shift in $T_{g2}$ from 174.3 °C to 194.17 °C. Notably, for the over-hydrolyzed sample P(St-MMA)−5, the distinct dual-$T_{g}$ behavior vanishes, replaced by a single broad transition at 119.3 °C. This thermal fusion implies that excessive hydrolysis destroys the boundaries of discrete microphase separation, resulting in a continuous, highly restricted ionomeric network where the pristine hydrophobic domains are completely overshadowed by the dominant ionic matrix relaxation.

### 3.2. Application-Guided Optimization of the Magnetic Layer Fabrication

The uniform and robust deposition of $Fe_{3}O_{4}$ nanoparticles onto the P(St-MMA) cores is pivotal for maintaining stable magnetic responsiveness and high immunoassay sensitivity. To this end, we systematically optimized the interfacial co-precipitation conditions, utilizing the ultimate RLU of the CEA CLEIA as a practical performance criterion.

#### 3.2.1. Effect of Substrate Hydrolysis Degree

**Figure 4:**
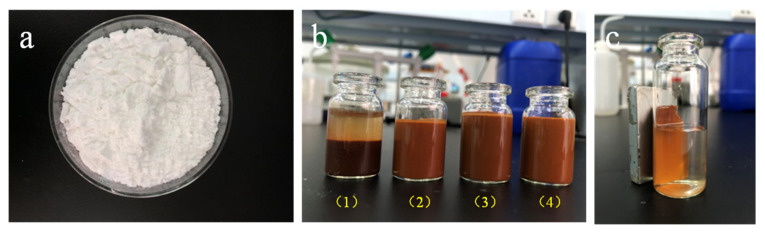
Digital photographs of (**a**) pristine P(St-MMA) powder, (**b**) $P(\mathrm{St}\text{-}\mathrm{MMA})@Fe_{3}O_{4}$ suspensions prepared with different degrees of substrate hydrolysis: (1)–(4) is P(St-MMA)−1 to −4@Fe_3_O_4_, and (**c**) the magnetic separation process.

The degree of alkaline hydrolysis profoundly dictated the $Fe_{3}O_{4}$ anchoring efficiency. As shown in Figure 4 and Figure S3, the insufficiently hydrolyzed sample (P(St-MMA)−1) exhibited a black appearance and rapid sedimentation. This indicates that the scarcity of surface carboxyl groups failed to provide sufficient coordination sites for iron ions, leading to severe bare-particle aggregation. Although the insufficiently hydrolyzed P(St-MMA)−1 seemingly exhibited an exceptionally high RLU in the presence of the antigen (Figure S3c), further analysis of the non-specific adsorption rate unambiguously proved this to be an artifact. Specifically, when evaluated in the blank control (0 ${ng\cdot mL}^{-1}$ CEA), the P(St-MMA)−1 sample generated an abnormally massive background signal (RLU > 150,000), indicating an extremely severe non-specific adsorption phenomenon. This is fundamentally attributed to the insufficient alkaline hydrolysis, which leaves a large area of highly hydrophobic styrene (St) segments exposed on the microsphere surface. These unhydrolyzed hydrophobic patches heavily promote the non-specific physical adsorption of enzyme-labeled tracer antibodies and matrix proteins. This severe non-specific binding, compounded by the physical trapping within the macroscopic aggregates, inherently distorted the true specific chemiluminescence response. Conversely, the fully hydrolyzed samples (P(St-MMA)−2 to −4) possessed abundant surface carboxylates, which tightly complexed with the $Fe_{3}O_{4}$ nanoparticles via robust coordination bonds. Consequently, these microspheres demonstrated excellent colloidal stability, slower sedimentation rates, and highly reproducible CLEIA responses.

To fundamentally elucidate this surface chemistry evolution and the underlying mechanism of the exceptional colloidal stability, Zeta potential measurements were conducted for the key fabrication stages (summarized in Table 5). The pristine P(St-MMA) microspheres exhibited a relatively low Zeta potential of −14.2 mV, primarily driven by sparse residual initiator fragments. Following the optimal alkaline hydrolysis (P(St-MMA)−4), the Zeta potential drastically decreased to −55.7 mV. This substantial enhancement in negative surface charge unambiguously confirms the massive generation and full deprotonation of carboxylate (-COO^−^)groups, which impart robust electrostatic repulsion to prevent core agglomeration during the severe co-precipitation conditions.

Subsequently, after the deposition of the inner magnetic layer, the Zeta potential of the $P(St\text{-}MMA)@Fe_{3}O_{4}$ intermediate shifted to −37.1 mV. This absolute value reduction indicates the successful coordination between the surface carboxylate sites and the iron precursor ions ($Fe^{2+}/Fe^{3+}$), as well as the charge-shielding effect of the deposited metal oxide. Following the final outer polymeric encapsulation, the $P(St\text{-}MMA)@Fe_{3}O_{4}@P(St\text{-}MMA)$ sandwich microspheres maintained a highly negative Zeta potential of −40.4 mV. In classical colloid and surface chemistry, an absolute Zeta potential exceeding 30 mV signifies a highly stable colloidal system driven by strong electrostatic repulsion [23]. This highly negative surface charge, combined with the steric hindrance provided by the outer amphiphilic polymer network, engenders a profound electro-steric stabilization effect. This physicochemical synergy perfectly rationalizes the exceptional long-term macroscopic stability (over 6 months without distinct sedimentation) observed in our study.

As a result, while the poorly hydrolyzed P(St-MMA)−1 yielded an artifactual signal spike due to massive agglomeration, the highly hydrolyzed P(St-MMA)−4 sample successfully avoided any macroscopic clustering. Thanks to its robust electro-steric stabilization and abundant accessible carboxyl anchoring sites, P(St-MMA)−4 delivered the highest true specific chemiluminescence response alongside the lowest background noise. Therefore, P(St-MMA)−3 was unambiguously identified as the optimal substrate and utilized exclusively for the subsequent fabrication of all $P(St-MMA)@Fe_{3}O_{4}$ and final sandwich-structured microspheres in this study.

#### 3.2.2. Screening of Dispersant Type and Concentration

**Figure 5:**
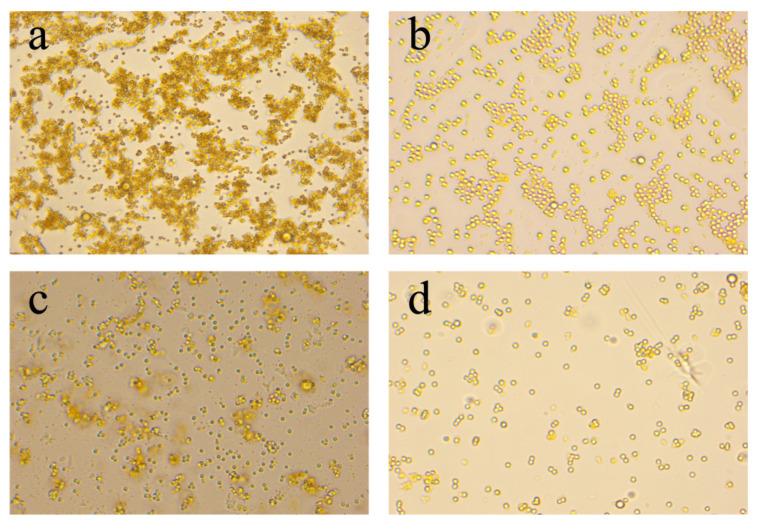
Optical microscopy images (1000×) of the $P(\mathrm{St}\text{-}\mathrm{MMA})@Fe_{3}O_{4}$ microspheres synthesized using various types of dispersants: (**a**) None, (**b**) (NH_4_)CO_3_, (**c**) Na_5_P_3_O_10_, and (**d**) K_2_HPO_4._

Apart from the substrate hydrolysis degree, the selection and concentration of the dispersant play a pivotal role. Given the inherent tendency of co-precipitated $Fe_{3}O_{4}$ nanoparticles to agglomerate, the introduction of a dispersant is imperative. Among the evaluated candidates (Figure 5 and Figure S4), ammonium carbonate ($(NH_{4}{)}_{2}CO_{3}$) emerged as the optimal dispersant. Not only did it yield a narrow size distribution and decelerated sedimentation rate but it also delivered an excellent CEA-Ag dose-response curve (Figure S4c). Crucially, $(NH_{4}{)}_{2}CO_{3}$ is thermally labile and decomposes into volatile gases (ammonia, $CO_{2}$, and water vapour), leaving no residual solids that might perturb the subsequent polymer encapsulation.

We further fine-tuned its concentration (Figure S5). The optimal dosing was identified at 6 wt%, which provided sufficient electrostatic/steric stabilization. In contrast, excessive dispersant concentrations (>12 wt%) unexpectedly accelerated sedimentation and broadened the size distribution (Figure S5a,b). This deterioration in colloidal stability is likely governed by the depletion flocculation mechanism, wherein an over-saturation of dispersant molecules compresses the electrical double layer and triggers nanoparticle aggregation.

#### 3.2.3. Influence of Magnetic Content and Buffer pH

The ratio of the magnetic components and the reaction pH were subsequently optimized. As depicted in Figure S6, a low theoretical magnetic content (20%) produced small microspheres with sluggish sedimentation. However, their weak magnetic susceptibility led to significant mass loss during the repeated magnetic washing steps of the immunoassay, ultimately resulting in a suppressed RLU (false negative trend). Conversely, an excessively high magnetic content (29%) also yielded a poor RLU. Mechanistically, an over-deposition of $Fe_{3}O_{4}$ nanoparticles heavily masks the active carboxyl sites on the P(St-MMA) surface. This steric shielding drastically hinders the subsequent EDC-mediated antibody conjugation. A magnetic content of 23–26% offered the optimal trade-off between magnetic separation efficiency and surface functionality.

Furthermore, the co-precipitation pH primarily dictated the thermodynamic driving force for $Fe_{3}O_{4}$ formation [24] and the deprotonation state of the surface carboxylate groups, which in turn profoundly influenced the magnetic loading efficiency and the colloidal stability of the composite microspheres (Figure S7). Extremely high or low pH environments disrupted the delicate nucleation-growth balance, leading to undesired aggregation. Specifically, as documented in classical co-precipitation studies [24], pH environments below 9 are highly prone to inducing the formation of non-superparamagnetic impurities (such as $\epsilon -Fe_{2}O_{3}$) rather than high-purity $Fe_{3}O_{4}$, thereby severely compromising the structural integrity and subsequent immunoassay performance.

**Figure 6:**
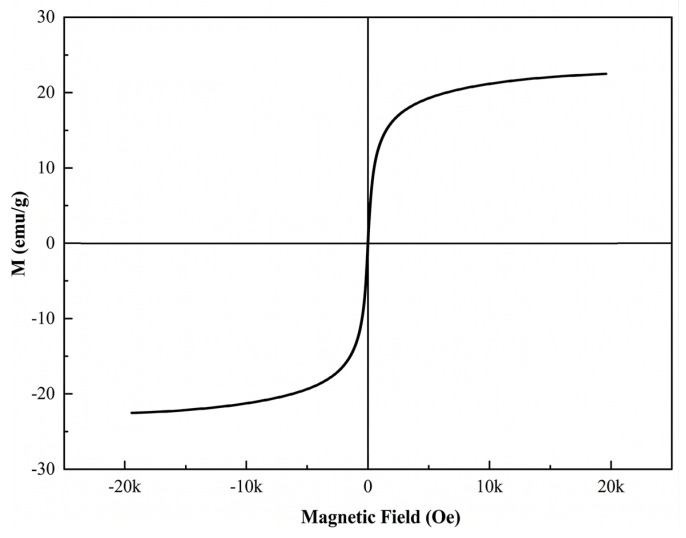
Room-temperature (300 K) magnetic hysteresis loop of the final sandwich-structured $P(St\text{-}MMA)@Fe_{3}O_{4}@P(St\text{-}MMA)$ magnetic polymeric microspheres.

To quantitatively evaluate the intrinsic magnetic responsiveness and verify the superparamagnetic behavior of the solid-phase carriers, VSM measurements were performed at 300 K. Figure 6 illustrates the field-dependent magnetization curve of the final optimized $P(St\text{-}MMA)@Fe_{3}O_{4}@P(St\text{-}MMA)$ sandwich microspheres. Critically, the curve exhibits a classical symmetric S-shape passing exactly through the origin. The complete absence of a distinct hysteresis loop, combined with virtually zero coercivity ($H_{c}$) and remanent magnetization ($M_{r}$), provides unambiguous evidence for the typical superparamagnetic characteristic of the engineered final materials, aligning with the expected magnetic behavior of isolated $Fe_{3}O_{4}$ cores [25].

Furthermore, the saturation magnetization ($M_{s}$) of the optimized three-layer sandwich microspheres was determined to be 22.6 ${emu\cdot g}^{-1}$. In the context of practical clinical diagnostics, an $M_{s}$ value exceeding 10 ${emu\cdot g}^{-1}$, coupled with absolute superparamagnetism, is highly sufficient. It not only achieves rapid and complete magnetic separation within 30 s under a standard external magnetic field but also fundamentally avoids irreversible magnetic agglomeration during the iterative incubation and washing cycles of fully automated CLEIA analyzer platforms.

### 3.3. Morphology, Porosity, and Long-Term Stability of the Three-Layer Sandwich Architecture

#### 3.3.1. Selection of the Protective Polymeric Shell

**Figure 7:**
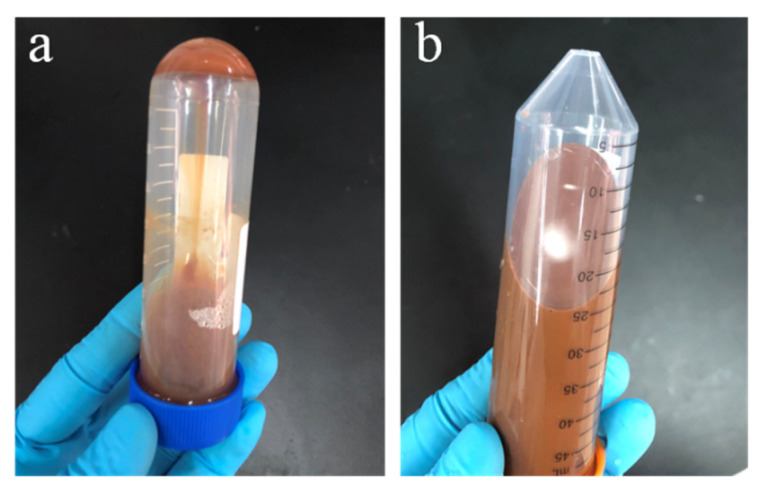
Visual observation of macroscopic aggregation and colloidal stability after 6 months of continuous buffer storage: (**a**) unprotected $P(\mathrm{St}\text{-}\mathrm{MMA})@Fe_{3}O_{4}$ and (**b**) sandwich-structured $P(\mathrm{St}\text{-}\mathrm{MMA})@Fe_{3}O_{4}@P(\mathrm{St}\text{-}\mathrm{MMA})$ microspheres.

Conventional magnetic polymeric microspheres intrinsically suffer from the direct exposure of outer $Fe_{3}O_{4}$ nanoparticles to the surrounding aqueous buffers. This vulnerability inevitably triggers progressive oxidation and massive agglomeration over prolonged storage, ultimately generating unintended deviations in CLEIAs. To construct a robust physical barrier against water and oxygen permeation, a secondary polymer encapsulation was strategically performed. Among the screened shell materials—PAA, PMMA, and P(St-MMA)—the PAA and PMMA coatings afforded high RLU values due to their abundant surface carboxyl groups, yet their inherent high hydrophilicity hindered uniform heterogeneous nucleation onto the magnetic cores, resulting in excessive particle sizes. Conversely, the P(St-MMA) copolymer, possessing a tailored amphiphilic balance, effectively minimized the interfacial tension. This favorable interfacial compatibility is primarily driven by the ‘like-wets-like’ principle, which minimizes the interfacial energy. Since both the inner core template and the newly grown outer shell share the identical P(St-MMA) chemical composition, the interfacial tension is drastically reduced. As indirectly evidenced by the morphological observations (which showed no secondary nucleation of free polymer particles) and the exceptional colloidal stability (validated by the highly negative Zeta potential of −40.4 mV and the 6-month macroscopic stability), this energetic favorability facilitated the formation of a highly uniform and continuous protective shell. Consequently, it ensured a compact final particle size and a significantly decelerated sedimentation rate. Strikingly, compared to the unprotected $P(\mathrm{St}\text{-}\mathrm{MMA})@Fe_{3}O_{4}$ microspheres, the sandwich-structured $P(\mathrm{St}\text{-}\mathrm{MMA})@Fe_{3}O_{4}@P(\mathrm{St}\text{-}\mathrm{MMA})$ microspheres exhibited exceptional long-term colloidal stability, completely resisting visible macroscopic aggregation even after 6 months of continuous storage in buffer solutions (Figure 7 and Figure S8).

It is worth noting that while direct spectroscopic investigations (such as XPS or XRD monitoring over time) are typically utilized to track the micro-chemical oxidation states of iron, the unprecedented 6-month macroscopic stability provides compelling functional and physical evidence of the proposed “oxidation-shielding” mechanism. Fundamentally, the progressive oxidation of $Fe_{3}O_{4}$ to $Fe_{2}O_{3}$ in aqueous environments inevitably triggers severe hydration, visual color alteration, and irreversible massive agglomeration. As clearly demonstrated in Figure 7, the sandwich-structured microspheres completely evaded these degradation phenomena. Furthermore, since severe oxidation dramatically degrades saturation magnetization, the sustained ability of the aged sandwich microspheres to undergo rapid magnetic separation (<30 s) during the extensive CLEIA operations serves as robust operational proof. This confirms that the outer hydrophobic P(St-MMA) shell acts as an impenetrable thermodynamic barrier against dissolved oxygen and water permeation, effectively preserving the structural and magnetic integrity of the inner $Fe_{3}O_{4}$ layer over prolonged storage.

#### 3.3.2. Optimization of P(St-MMA) Encapsulation Amount

**Figure 8:**
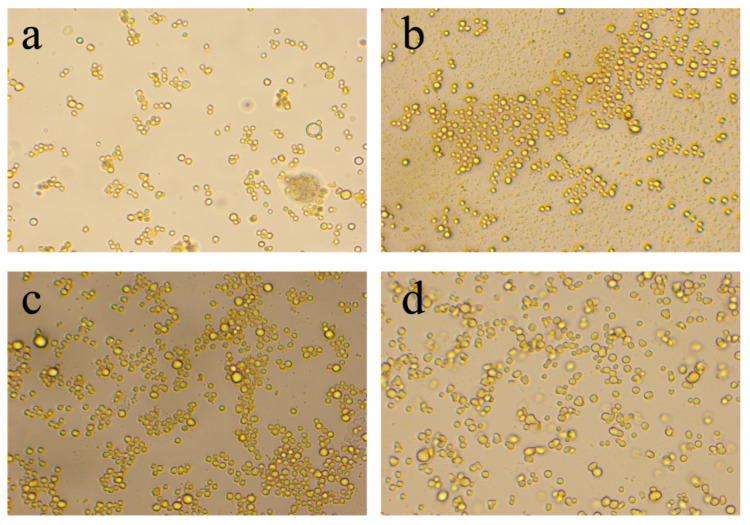
Optical microscopy images (1000×) of the $P(\mathrm{St}\text{-}\mathrm{MMA})@Fe_{3}O_{4}@P(\mathrm{St}\text{-}\mathrm{MMA})$ microspheres engineered with varying amounts of outer P(St-MMA) encapsulation: (**a**) 30%, (**b**) 50%, (**c**) 70%, and (**d**) 80%.

The thickness of the outer P(St-MMA) shell was further optimized by varying the monomer feed ratio (Figure 8 and Figure S9). An insufficient coating amount (e.g., 30%) failed to fully cover the magnetic layer, while excessive amounts (70% and 80%) induced uneven shell overgrowth, leading to an enlarged particle size and accelerated sedimentation. The optimal encapsulation amount was identified at 50%, which yielded monodisperse sandwich microspheres with a highly uniform average diameter of 1.7 μm (PDI = 0.24) and maximized the chemiluminescence RLU response.

#### 3.3.3. Porosity and Textural Properties

Nitrogen adsorption-desorption measurements were conducted to elucidate the textural evolution of the microspheres (Figure S10 and Table 6). According to the IUPAC classification, both the $P(\mathrm{St}\text{-}\mathrm{MMA})@Fe_{3}O_{4}$ and sandwich-structured microspheres exhibited characteristic Type IV isotherms with distinct hysteresis loops at moderate to high relative pressures ($P/P_{0}$), which unambiguously corroborates the presence of a well-defined mesoporous architecture. The uncoated $P(\mathrm{St}\text{-}\mathrm{MMA})@Fe_{3}O_{4}$ possessed a specific surface area of 18.811 $m^{2}\cdot g^{-1}$ and an average pore size of 17.52 nm. These primary mesopores likely originate from the interstitial voids between the closely packed $Fe_{3}O_{4}$ nanoparticles on the core surface. Upon the 50% outer P(St-MMA) encapsulation, the specific surface area and total pore volume predictably decreased to 9.552 $m^{2}\cdot g^{-1}$ and 0.063 $m^{3}\cdot g^{-1}$, respectively, accompanied by a reduction in the average pore size to 13.11 nm. This textural attenuation further solidifies the successful infusion and coating of the outer P(St-MMA) chains into the superficial magnetic voids, structurally locking the $Fe_{3}O_{4}$ nanoparticles and providing an indispensable defensive shield for practical clinical diagnostics.

Furthermore, while the direct observation of the internal cross-sectional architecture of polymer/inorganic hybrids can be technically challenging due to beam sensitivity, the successful formation of the $P(St\text{-}MMA)@Fe_{3}O_{4}@P(St\text{-}MMA)$ sandwich structure is unambiguously corroborated by a multi-dimensional evidence chain in this study. The progressive increase in microsphere diameter (SEM), the emergence of specific thermal phase transitions (DSC), and, critically, the significant attenuation in specific surface area and pore volume (BET) collectively confirm the sequential deposition and sealing of the outer P(St-MMA) shell. Ultimately, the unprecedented 6-month macroscopic colloidal stability against oxidation (Figure 7) serves as the most definitive functional proof of this protective sandwich architecture.

Beyond structural confirmation, this well-defined mesoporous architecture plays a pivotal role in dictating immunoassay performance. The structure-function relationship is highly dependent on the “size-matching effect” between the mesopores and the biomolecules. Typical IgG capture antibodies exhibit hydrodynamic dimensions of approximately 10–15 nm [26]. Remarkably, the average pore size of the optimized sandwich microspheres is 13.11 nm (Table 6), which perfectly matches the spatial footprint of the CEA antibodies. Instead of merely adsorbing onto a flat exterior, these engineered mesopores act as nanoscale ‘cradles’, which is a highly favored topological matching strategy for preventing the steric burying of biomacromolecules [27]. They allow the antibodies to be firmly anchored via multi-point EDC/NHS covalent bonding while preventing them from being trapped deeply within inaccessible internal voids. Furthermore, the extensive specific surface area and the rough mesoporous topology provide an abundance of accessible carboxylate (−COOH) anchoring sites. The localized curvature within these mesopores also helps to properly space the immobilized antibodies, thereby mitigating steric hindrance between adjacent biomolecules and exposing their Fab fragments favorably toward the surrounding solution. Consequently, this optimal spatial orientation and enhanced antibody loading efficiency directly translate into superior antigen capture kinetics, which fundamentally underlies the exceptionally high chemiluminescence response and the ultrasensitive detection limit achieved in the final CLEIA application.

#### 3.3.4. Amphiphilic Synergy for Shielding and Sensing

**Figure 9:**
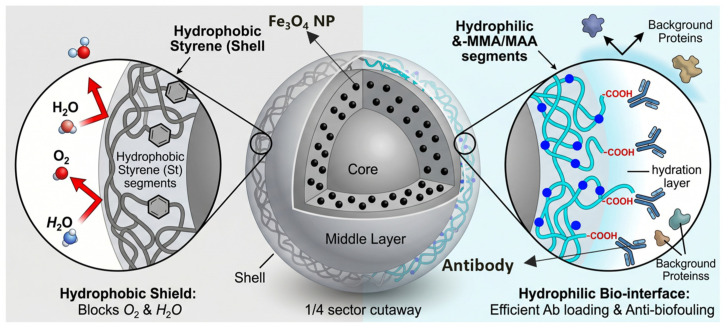
Schematic mechanism illustrating the amphiphilic synergy of the outer P(St-MMA) shell. The hydrophobic styrene (St) segments provide an impermeable shield against oxidation ($O_{2}$/$H_{2}O$ exclusion), while the hydrophilic MMA/MAA segments establish a robust bio-interface for highly efficient antibody conjugation and the suppression of non-specific binding.

The exceptional overall performance of the $P(St\text{-}MMA)@Fe_{3}O_{4}@P(St\text{-}MMA)$ microspheres is fundamentally governed by the amphiphilic nature of the outer polymeric shell (Figure 9). This bespoke amphiphilic architecture uniquely unifies two seemingly contradictory requirements: stringent internal protection and highly reactive external bioconjugation.

On the one hand, the hydrophobic styrene (St) segments constitute a robust physical and thermodynamic shield. The intrinsic hydrophobicity and spatial steric hindrance of the aromatic rings severely restrict the permeation of water molecules and dissolved oxygen from the surrounding environment. This structural impermeability hermetically seals the intermediate $Fe_{3}O_{4}$ nanoparticles, completely suppressing progressive oxidation and ensuring long-term magnetic and colloidal stability.

On the other hand, the hydrophilic methyl methacrylate (MMA) and its subsequently hydrolyzed methacrylic acid (MAA) segments predominantly orient toward the solid-liquid interface. This hydrophilic configuration creates a stable surface hydration layer that minimizes non-specific protein adsorption, significantly depressing the background noise during the chemiluminescence readout. More importantly, these externally exposed segments provide an ultra-high density of accessible carboxylate (−COOH) anchoring sites. This guarantees an exceptionally high loading efficiency of the CEA capture antibodies via stable EDC/NHS covalent bonding, ultimately maximizing the antigen-capture kinetics and assay sensitivity. Thus, the synergistic integration of hydrophobic shielding and hydrophilic functionalization within a single polymeric shell essentially dictates the success of these bespoke solid-phase carriers in ultrasensitive CLEIA applications.

### 3.4. Analytical Performance and Commercial Comparison

To rigorously validate the clinical diagnostic capability of the optimized $P(St\text{-}MMA)@Fe_{3}O_{4}@P(St\text{-}MMA)$ sandwich microspheres, comprehensive analytical parameters were evaluated and benchmarked against industry-standard commercial beads (JSR Life Sciences Magnosphere^TM^ MS160/Carboxyl).

**Figure 10:**
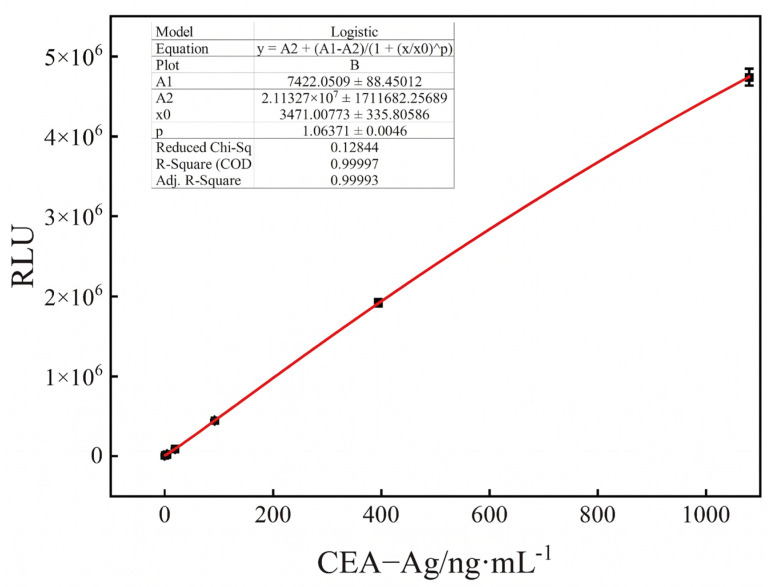
Four-parameter logistic calibration curve for the CEA CLEIA using the engineered sandwich microspheres.

A standard curve for CEA detection was established using a four-parameter logistic fitting model (Figure 10). The calibration curve exhibited an excellent correlation coefficient ($R^{2}$) of 0.99997 over the tested dynamic range, demonstrating exceptional linear fitting performance. Based on the mean background signal plus three times the standard deviation, the limit of detection (LOD) was determined to be 0.055 ${ng\cdot mL}^{-1}$. Furthermore, analytical recovery was assessed by spiking known concentrations of CEA-Ag. As summarized in Table 7, the average recovery rates ranged from 93.37% to 108.25% across different concentration levels, indicating high diagnostic accuracy and negligible matrix interference.

To further assess the reproducibility and practical translational potential, precision and inter-assay variations were compared head-to-head with the commercial JSR beads. As shown in Table 8, the intra-assay precision for the homemade microspheres yielded coefficients of variation (CV) of 4.37%, 3.17%, and 4.25% for low-, medium-, and high-concentration samples, respectively. These values are consistently below 5% and highly comparable to those of the commercial counterparts. Similarly, the inter-assay variations across three independent batches (Table 9) exhibited minimal fluctuation, with CVs well below the 10% clinical acceptability threshold. Crucially, under identical operational conditions, the engineered sandwich microspheres outperformed the commercial JSR beads by delivering stronger chemiluminescent signals (RLU) across all positive antigen concentrations, while simultaneously suppressing the background noise (0 ng/mL) to a lower level. This synergistic enhancement in both signal amplification and noise reduction underscores the profound advantages of the bespoke mesoporous shielding architecture, highlighting its immense potential as a robust solid-phase carrier in next-generation IVD.

## 4. Conclusions

In summary, a facile, highly controllable, and application-guided strategy was successfully developed to fabricate monodisperse magnetic polymeric microspheres featuring a precisely defined $P(\mathrm{St}\text{-}\mathrm{MMA})@Fe_{3}O_{4}@P(\mathrm{St}P(St\text{-}MMA)\mathrm{MMA})$ three-layer sandwich architecture. By systematically optimizing the interfacial co-precipitation parameters, we demonstrated that adequate alkaline hydrolysis of the substrate, coupled with the introduction of 6 wt% thermally labile $(NH_{4}{)}_{2}CO_{3}$ as a clean dispersant, effectively suppressed the non-specific aggregation and false-positive signaling commonly encountered in conventional magnetic beads. Furthermore, the tailored 50% P(St-MMA) outer shell functioned as an indispensable oxidation and hydration shield while preserving an accessible mesoporous network (average pore size of ~13.11 nm).

Crucially, this rationally designed sandwich structure endowed the microspheres with exceptional long-term colloidal stability, entirely preventing macroscopic aggregation for over 6 months in aqueous buffer systems. When directly integrated into a commercial CEA CLEIA diagnostic kit, the optimized microspheres exhibited outstanding bioconjugation capacity and highly sensitive, reproducible RLU responses.

Ultimately, this work offers a robust and scalable materials platform for advancing solid-phase carriers in clinical IVD. Future structural refinements will be directed toward elucidating the precise interfacial binding mechanisms between the magnetic cores and the amphiphilic polymer chains through molecular dynamics simulations. By extracting radial distribution function (RDF) peaks and mean square displacement (MSD) parameters, we anticipate gaining profound theoretical insights into interfacial mobility and thermodynamic compatibility, which will further accelerate the industrial translation of these advanced functional materials for next-generation high-throughput immunoassay systems.

## Figures and Tables

**Table 1:** Comparison of previously reported magnetic polymeric microspheres and the proposed sandwich-structured microspheres for immunoassay applications.

Material/Architecture	Pore Size (nm)	Oxidation Resistance	Colloidal Stability	Immunoassay Application	Ref.
Fe_3_O_4_@PS (Core-Shell)	Not specified/Low	Moderate (Exposed surface)	Moderate (Prone to aggregation)	Food safety detection	[14]
Dextran-grafted magnetic polymer	Not specified	Moderate	Good	CLEIA carriers	[18]
PGMA magnetic microspheres	Non-porous	Weak (Susceptible in buffer)	Moderate	Autoantibody isolation	[19]
Epoxy magnetic beads	Low	Moderate	Good	Monoclonal antibody quantitation	[20]
$P(\mathrm{St}\text{-}\mathrm{MMA})@Fe_{3}O_{4}@P(\mathrm{St}\text{-}\mathrm{MMA})$ (Sandwich)	13.11 (Mesoporous)	Excellent (Shielded)	Exceptional (>6 Months)	Ultrasensitive CEA CLEIA	This work

**Table 2:** Hydrolysis parameters and particle size characteristics of the pristine and alkaline-hydrolyzed P(St-MMA) microspheres.

Sample	Sample Mass/g	NaOH Concentration	Hydrolysis Cycles	Average Particle Size/µm	Polydispersity Index/PDI
P(St-MMA)	58	/	/	0.95 ± 0.00	0.13 ± 0.00
P(St-MMA)−1	58	13	1	6.15 ± 0.07	0.41 ± 0.02
P(St-MMA)−2	58	15	3	1.37 ± 0.01	0.12 ± 0.01
P(St-MMA)−3	58	13	3	1.34 ± 0.02	0.43 ± 0.02
P(St-MMA)−4	115	8	6	1.54 ± 0.14	1.46 ± 0.18
P(St-MMA)−5	115	33	3	18.61 ± 0.00	0.16 ± 0.01

Note: P(St-MMA)−4 was identified as an unstable/failed sample due to excessive hydrolysis, resulting in severe particle aggregation and a high PDI.

**Table 3:** Detailed FTIR band assignments for the pristine and alkaline-hydrolyzed P(St-MMA) microspheres.

Wavenumber (cm^−1^)	Functional Group/Vibration Mode	Origin (Polymer Segment)
3436	O–H stretching (broad)	Adsorbed water/Carboxyl groups (from MAA)
3023	C–H stretching (aromatic ring)	Styrene (St)
2944, 2851	C–H stretching (aliphatic −CH_2_− and −CH_3_)	Main chain and MMA/MAA
1734	C=O stretching (sharp)	Ester groups (from MMA)
1598, 1498, 1451	C=C skeleton stretching	Aromatic ring (from St)
1381	C–H bending (in-plane, methyl group)	Methyl groups
1195	C–O–C stretching	Ester bonds (from MMA)

**Table 4:** Thermal properties (DSC results) of the pristine and alkaline-hydrolyzed P(St-MMA) microspheres.

Sample Name	*T*_*g*1_/°C	*T*_*g*2_/°C
P(St-MMA)	95.1	/
P(St-MMA)−1	105.2	/
P(St-MMA)−2	105.9	174.3
P(St-MMA)−3	105.1	178.9
P(St-MMA)−4	105.1	194.17
P(St-MMA)−5	119.3	/

Note: $T_{g1}$ and $T_{g2}$ denote the first and second glass transition temperatures, respectively.

**Table 5:** Zeta potential variations of the microspheres at different key fabrication stages.

Sample Stage	Sample Name	Zeta Potential/mV
Pristine Core	P(St-MMA)	−14.2 ± 0.8
Optimal Hydrolyzed Core	P(St-MMA)−4	−55.7 ± 1.2
Inner Magnetic Intermediate	$P(\mathrm{St}\text{-}\mathrm{MMA})@Fe_{3}O_{4}$	−37.1 ± 1.5
Final Sandwich Microspheres	$P(\mathrm{St}\text{-}\mathrm{MMA})@Fe_{3}O_{4}@P(\mathrm{St}\text{-}\mathrm{MMA})$	−40.4 ± 1.1

**Table 6:** Textural properties (BET specific surface area, pore volume, and pore size) of the uncoated and sandwich-structured magnetic microspheres.

P(St-MMA) Encapsulation Amount	a_s, BETmp_/$m^{2}\cdot g^{-1}$	Total Pore Volume/$m^{3}\cdot g^{-1}$	Average Pore Size/nm
None	18.811	0.165	17.520
30%	9.888	0.061	12.293
50%	9.552	0.063	13.115
70%	7.775	0.067	17.196
80%	5.398	0.058	21.288

**Table 7:** Analytical recovery of CEA-Ag using the engineered sandwich microspheres.

Specimen Concentration (ng/mL)	RLU	Measured Concentration/${ng\cdot mL}^{-1}$	Recovery/%	Average Recovery/%
3.8	23,853/23,095/22,998	3.69/3.49/3.46	97.21/91.80/91.10	93.37
18.52	88,426/86,313/88,027	20.25/19.74/20.15	109.32/106.61/108.81	108.25
91.66	430,252/459,213/440,841	89.05/94.41/91.01	97.16/103.00/99.30	99.82
394.68	1,914,746/1,965,077/1,870,942	369.88/380.34/360.85	93.72/96.37/91.43	93.84
1080.24	4,815,801/4,779,295/4,615,671	1171.20/1157.85/1099.29	108.42/107.18/101.76	105.79

**Table 8:** Intra-assay precision comparison between the engineered sandwich microspheres and commercial JSR beads.

Parameters	$P(\mathrm{St}\text{-}\mathrm{MMA})@Fe_{3}O_{4}@P(\mathrm{St}\text{-}\mathrm{MMA})$			JSR Magnosphere™ MS160/Carboxyl		
Sample ID	S2	S4	S6	S2	S4	S6
Average RLU	21,388	368,441	4,787,389	21,343	378,072	4,279,638
SD	935	11,677	203,594	994	14,371	181,197
CV (%)	4.37%	3.17%	4.25%	4.66%	3.80%	4.23%

**Table 9:** Inter-assay variation (batch-to-batch) comparison across three independent lots.

CEA-Ag (ng/mL)	Engineered Sandwich Microspheres/RLU			Commercial JSR Beads/RLU		
	Lot 1	Lot 2	Lot 3	Lot 1	Lot 2	Lot 3
0	7685	7809	7756	8064	8171	8229
3.8	21,258	20,317	20,324	18,912	18,564	17,382
18.52	87,607	90,158	80,940	71,005	70,352	67,465
91.66	433,165	456,518	431,952	362,012	358,402	371,316
394.68	1,940,568	1,973,907	1,872,844	1,657,801	1,577,764	1,705,096
1080.24	4,868,172	4,884,004	4,964,854	4,331,750	4,204,452	4,546,036

## Data Availability

The data presented in this study are available upon request from the corresponding author.

## Supplementary Materials

The following supporting information can be downloaded at https://www.mdpi.com/article/10.3390/bios16060303/s1, Figure S1: FTIR spectra of the pristine and alkaline-hydrolyzed P(St-MMA) microspheres; Figure S2: DSC thermograms of the pristine and alkaline-hydrolyzed P(St-MMA) microspheres; Figures S3 to S9: systematic parameter optimization profiles—including particle size distributions, sedimentation rates, CEA-Ag dose-response curves, and signal-to-noise ratios—evaluating the effects of substrate hydrolysis degree, dispersant concentration, magnetic component content, co-precipitation pH, and outer protective shell encapsulation; Figure S10: $N_{2}$ adsorption-desorption isotherms alongside pore size distribution curves.
