# Supplementary material for: Oxidation-Shielded P(St-MMA)@Fe3O4@P(St-MMA) Mesoporous Magnetic Microspheres: A Robust Solid-Phase Carrier for Ultrasensitive CEA Chemiluminescence Immunoassay
Source: Biosensors (Basel). 2026 May 22;16(6):303. doi: 10.3390/bios16060303 (PMC13297564; doi:10.3390/bios16060303)
Supplement: Supplementary file 1 [file biosensors-16-00303-s001.zip › biosensors-4291249-supplementary.pdf]

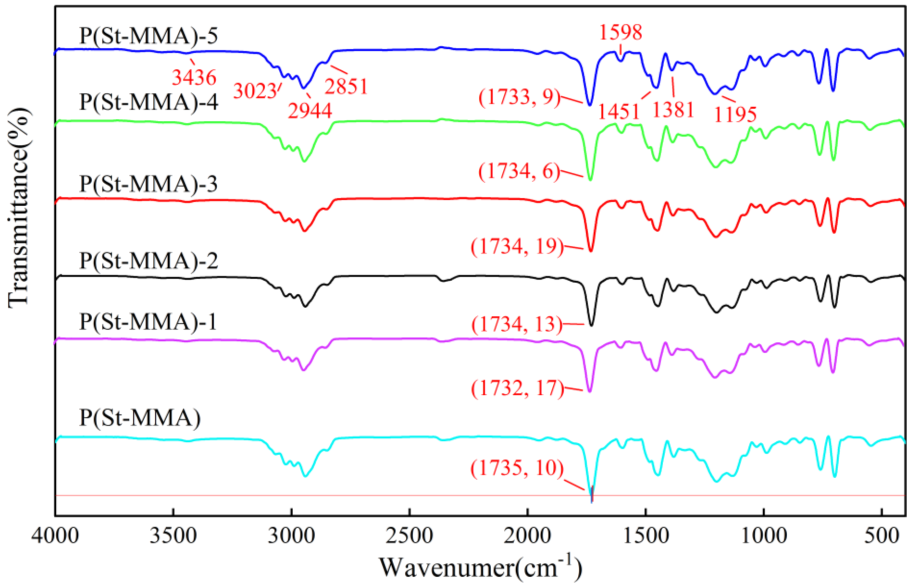

Figure S1. FTIR spectra of the pristine and alkaline-hydrolyzed P(St-MMA) microspheres.

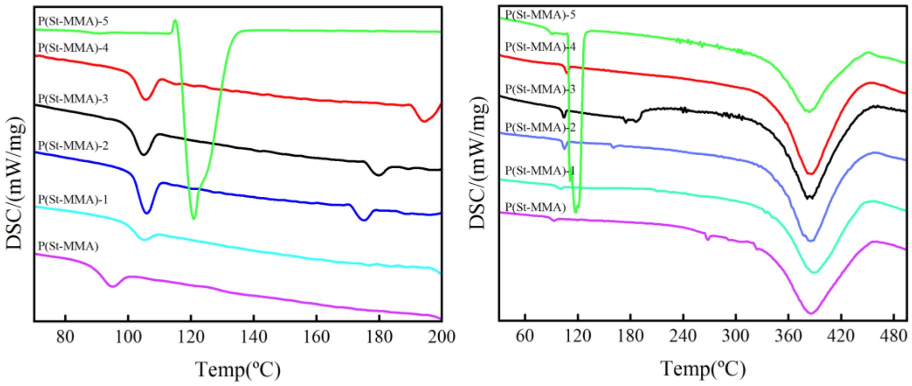

Figure S2. DSC thermograms of the pristine and alkaline-hydrolyzed P(St-MMA) Microspheres.

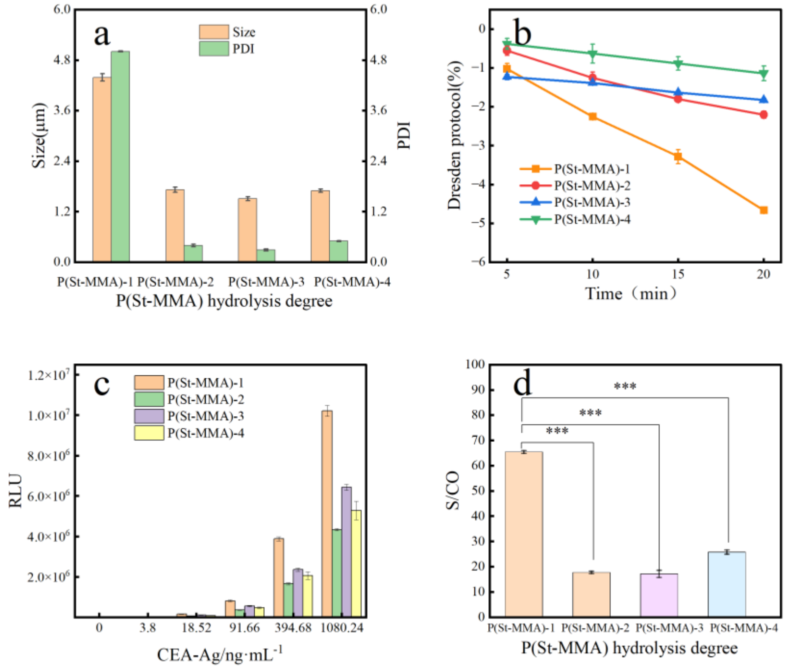

Figure S3. Effect of substrate hydrolysis degree on the Fe_3O_4 coating efficiency: (a) particle size distribution, (b) sedimentation profile monitored at OD_{560}, (c) CEA-Ag dose-response chemiluminescence curves, and (d)Signal-to-Noise Ratio. **Data are presented as mean ± standard deviation (SD) from three independent experiments (n = 3). Statistical significance was determined using Student's t-test (* p < 0.05, ** p < 0.01, *** p < 0.001).**

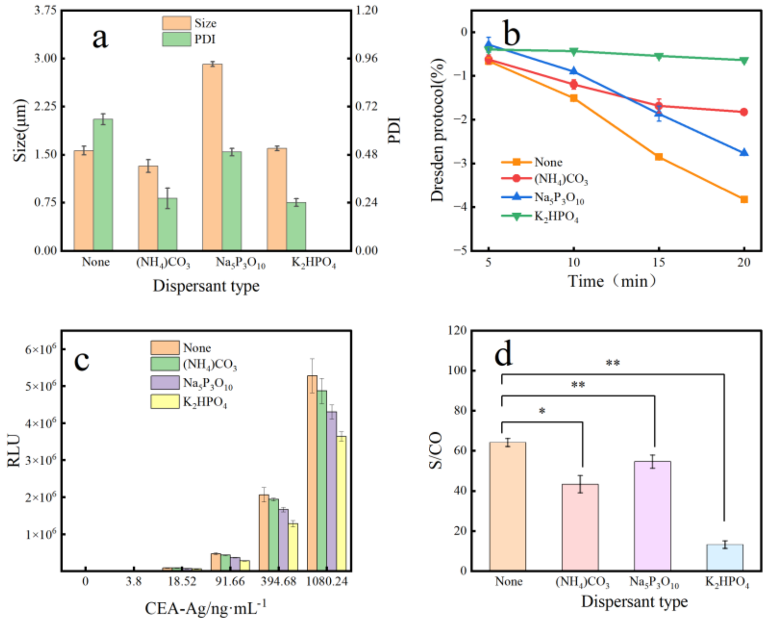

Figure S4. Effect of substrate hydrolysis degree on the Fe_3O_4 coating efficiency: (a) particle size distribution, (b) sedimentation profile monitored at OD_{560}, (c) CEA-Ag dose-response chemiluminescence curves, and (d)Signal-to-Noise Ratio. **Data are presented as mean ± standard deviation (SD) from three independent experiments (n = 3). Statistical significance was determined using Student's t-test (* p < 0.05, ** p < 0.01, *** p < 0.001).**

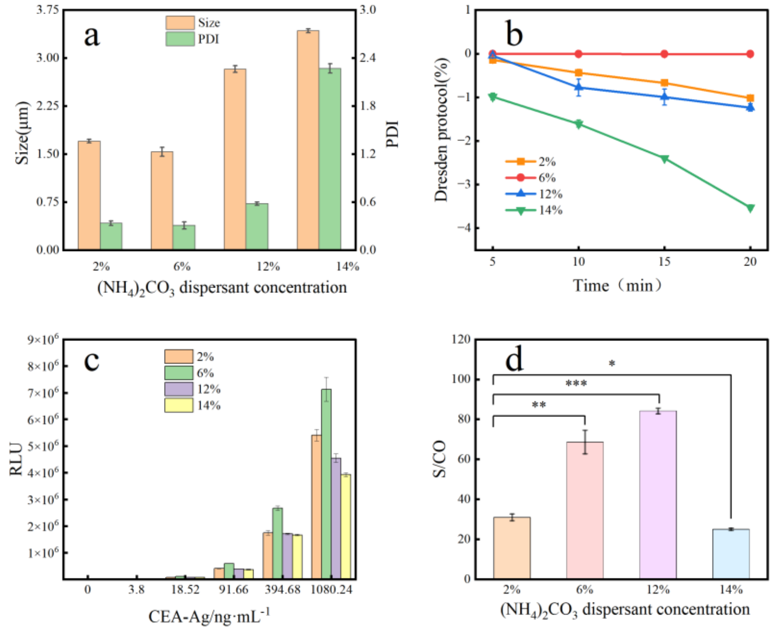

Figure S5. Effect of $(NH_4)_2CO_3$ dispersant concentration on the $P(St\text{-}MMA)@Fe_3O_4$ microspheres: (a) particle size distribution, (b) sedimentation profile at OD_{560}, (c) CEA-Ag dose-response chemiluminescence curves, and (d)Signal-to-Noise Ratio. **Data are presented as mean ± standard deviation (SD) from three independent experiments (n = 3). Statistical significance was determined using Student's t-test (* p < 0.05, ** p < 0.01, *** p < 0.001).**

https://doi.org/10.3390/bios16060303

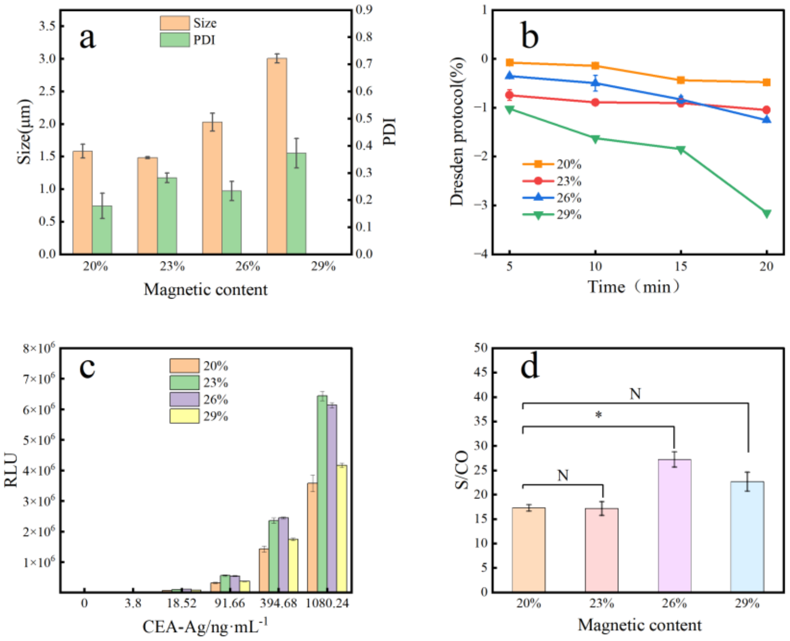

Figure S6. Influence of magnetic component content on the $P(St\text{-}MMA)@Fe_3O_4$ microspheres: (a) particle size distribution, (b) sedimentation profile at OD_{560}, (c) CEA-Ag dose-response chemiluminescence curves, and (d)Signal-to-Noise Ratio. **Data are presented as mean ± standard deviation (SD) from three independent experiments (n = 3). Statistical significance was determined using Student's t-test (* p < 0.05, ** p < 0.01, *** p < 0.001).**

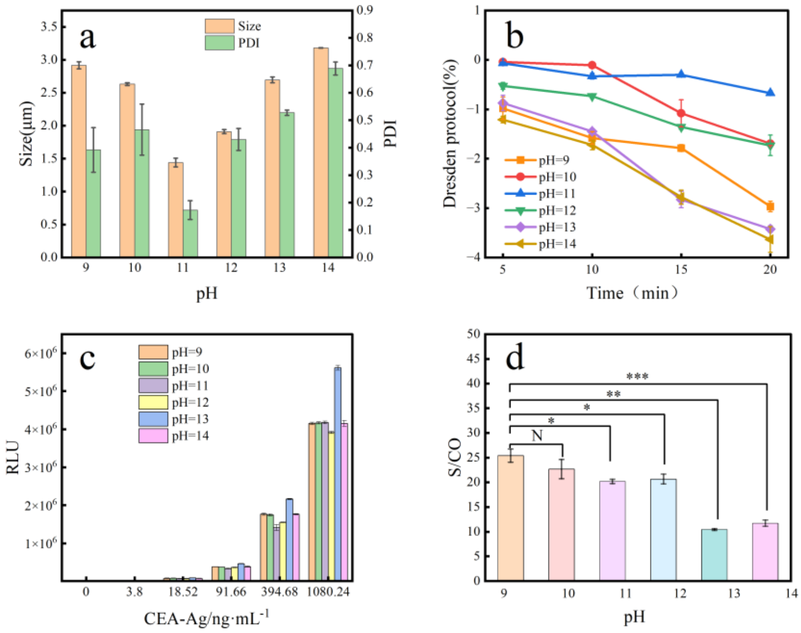

Figure S7. Effect of co-precipitation pH on the *P*(*St*-*MMA*)@*Fe*3*O*4 microspheres: (a) particle size distribution, (b) sedimentation profile monitored at *OD*560, (c) CEA-Ag dose-response chemiluminescence curves, and (d)Signal-to-Noise Ratio. **Data are presented as mean ± standard deviation (SD) from three independent experiments (n = 3). Statistical significance was determined using Student's t-test (* p < 0.05, ** p < 0.01, *** p < 0.001).**

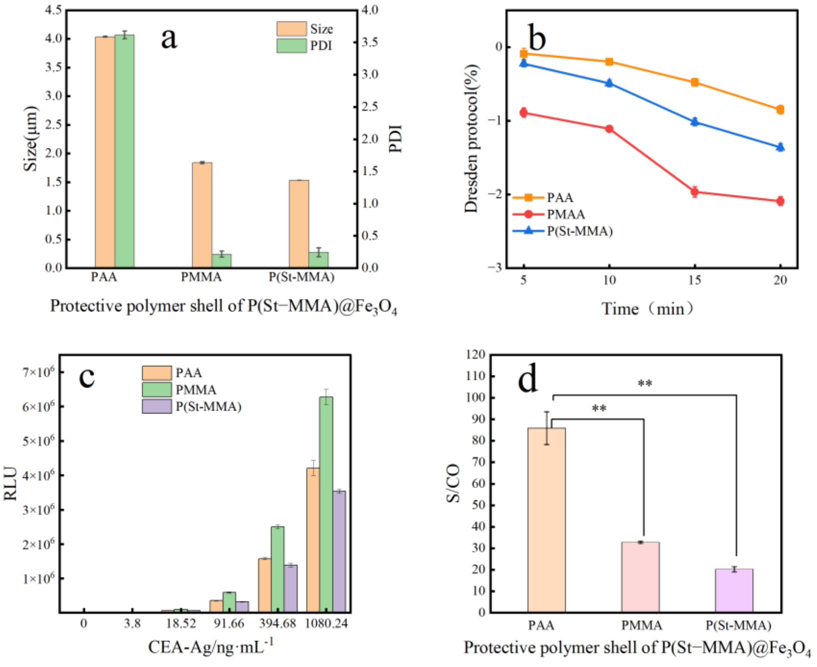

Figure S8. Comparison of different outer protective polymeric shells on the *P*(*St-MMA*)@*Fe*3*O*4 microspheres: (a) particle size distribution, (b) sedimentation profile monitored at *OD*560, (c) CEA-Ag dose-response chemiluminescence curves, and (d)Signal-to-Noise Ratio.

Data are presented as mean ± standard deviation (SD) from three independent experiments (n = 3). Statistical significance was determined using Student's t-test (* p < 0.05, ** p < 0.01, *** p < 0.001).

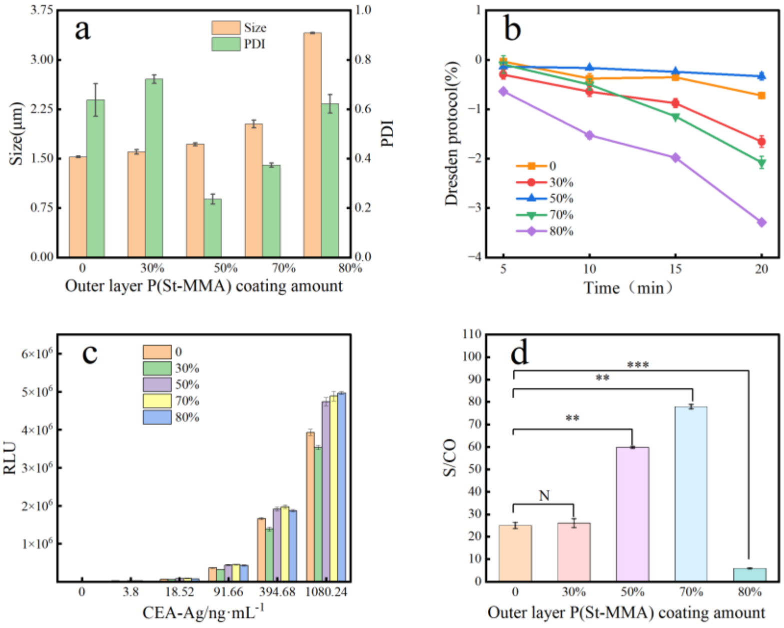

Figure S9. Optimization of the outer P(St-MMA) encapsulation amount: (a) particle size distribution, (b) sedimentation profile at OD_{560}, (c) CEA-Ag dose-response chemiluminescence curves, and (d)Signal-to-Noise Ratio.

https://doi.org/10.3390/bios16060303

Data are presented as mean ± standard deviation (SD) from three independent experiments (n = 3). Statistical significance was determined using Student's t-test (* $p < 0.05$, ** $p < 0.01$, *** $p < 0.001$).

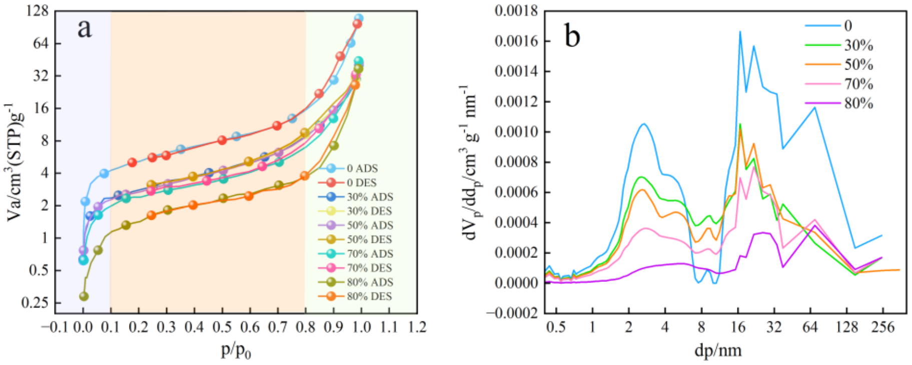

Figure S10. (a) N_2 adsorption-desorption isotherms and (b) pore size distribution curves of the uncoated and encapsulated microspheres.

Data are presented as mean ± standard deviation (SD) from three independent experiments (n = 3). Statistical significance was determined using Student's t-test (* $p < 0.05$, ** $p < 0.01$, *** $p < 0.001$).

Disclaimer/Publisher's Note: The statements, opinions and data contained in all publications are solely those of the individual author(s) and contributor(s) and not of MDPI and/or the editor(s). MDPI and/or the editor(s) disclaim responsibility for any injury to people or property resulting from any ideas, methods, instructions or products referred to in the content.

https://doi.org/10.3390/bios16060303
